# Creep-Induced Screw Preload Loss of Carbon-Fiber Sheet Molding Compound at Elevated Temperature

**DOI:** 10.3390/ma12213598

**Published:** 2019-11-01

**Authors:** David Finck, Christian Seidel, Joachim Hausmann, Thomas Rief

**Affiliations:** 1 SIEMENS AG, Corporate Technology REE MDM POL-DE, 91058 Erlangen, Germany; christian.seidel@siemens.com; 2 IVW – Institute for Composite Materials GmbH, Component Development, 67663 Kaiserslautern, Germany; joachim.hausmann@ivw.uni-kl.de (J.H.); thomas.rief@ivw.uni-kl.de (T.R.)

**Keywords:** creep, relaxation, screw, preload loss, composites, sheet mold compound, numerical analysis

## Abstract

The application of chopped-fiber reinforced polymers in screwed connections at high temperatures raises the question of creep under long-term loading. While up to now thermoplastic materials have mainly been the focus of attention when it comes to creep, this paper shows that thermoset carbon-fiber SMCs (sheet mold compounds) can also be affected by this phenomenon. Screwed connections were investigated regarding their loss of preload force at 120 °C ambient temperature. Additionally, strain–time diagrams were recorded at different stress levels at 120 °C in a creep test setup of a universal testing machine by using optical strain tracking of SMC coupons. The transverse modulus under compression in thickness direction was determined in the same test setup. For data application within a FEA (finite element analysis) software power law curves according to Norton–Bailey creep law were fitted in the strain–time graphs. The applicability of the obtained creep law was crosschecked with a test carried out on the loss of preload force of a screwed connection. The developed simulative methodology offers the possibility to simulate various mounting situations of the bolted connection and to investigate measures against the loss of preload force easily. A promising possibility to limit the loss of preload force due to creep was simulatively evaluated.

## 1. Introduction

Usually, a screw in mechanical engineering is used to clamp two materials with a force, which is high enough to induce friction and thus avoid sliding between the surfaces of the clamped parts. The factors influencing the sliding of the surfaces against each other are the clamping force of the screw and the coefficient of friction between the screwed surfaces. Attention in this study is put on the clamping force. The clamping force may not be too high, to avoid unnecessary high stress in the screw or yielding of the clamped material. If the clamping force decreases too much, the screwed surfaces can move relative to each other, due to an external force. Both limit cases should be avoided. Therefore, a change of screw preload force, after the tightening of the screw, is not desired or at least has to be within well-known limits [1,2].

Screw connections suffer from loss of preload, due to different reasons: dynamic forces can cause settlement of surface roughnesses, rotational self-loosening of the bolt/nut, and/or fatigue of the clamped/bolt material [1]. Plenty of research was done on this topic, because the failure of a screwed connection can lead to dramatic consequences. In the following, the focus is on the creep of the screwed material, which is caused by the bolt pretension. 

There are several approaches on prediction of preload loss for screw assemblies [3,4,5,6,7]. They are based on empirical analytical equations, that approximate the screw force preload over time. The accuracy and applicability of these empirical equations for various screw mounting situations is hard to predict. 

Alternatively, it is also possible to develop a universal creep law for a material in the FEA, which allows a simulative problem analysis in different application scenarios. This procedure, with full implementation in the FEA, has been reported for unreinforced epoxy specimens, i.e. isotropic material [8]. The creep of CFRP (carbon-fiber reinforced polymer) coupons in thickness direction was measured by [9], also with the aim of bolt preload loss prediction. However, no performable implementation into FE software followed. This way, the applicability of the creep power law to orthotropic materials in the FEA remained unclear, as creep laws are typically only useable with isotropic material cards in known commercial FE suits.

In this paper the work of [9] was picked up and the applicability of the Norton–Bailey creep power law to orthotropic C-SMC (carbon-fiber sheet molding compound) material loaded in the thickness direction was confirmed. Studies were carried out on one C-SMC from manufacturer Polynt at 120 °C ambient temperature. The temperature was selected due to a possible use of the mentioned material in mobility. The temperature of 120 °C is an often referred upper operating limit here [10]. Creep strain curves were recorded for the investigated C-SMC material and approximated with the Norton–Bailey creep law. The Norton–Bailey creep law is considered the simplest creep law and a standard at common FE suits like MSC Nastran, ABAQUS, ANSYS Workbench or SIEMENS NX Simcenter [11,12,13,14]. The use of other creep laws, further detail at e.g., [15], would also be conceivable. However, since curve fitting by use of the Norton–Bailey creep law achieved a sufficiently good approximation of the measurement data, it was selected here. Creepage of the C-SMC material was also tested in a screw preload test with a force measuring washer. The developed power law was successfully crosschecked with the screw preload test. The crosscheck with the screw preload experiment revealed also, that a far-reaching extrapolation of the obtained creep data is possible. The availability of a creep law in FEA software offers various possibilities. Different screw designs can easily be checked with respect to the loss of screw preload. A simulation of a real screw application, without distorting stiffness influence of measurement equipment, is presented. In addition, a solution for limiting preload losses at a bolted joint was investigated. 

## 2. Investigated SMC Material and Processing

SMC sheets typically contained chopped fiber-rovings, with lengths up to 50 mm with random orientations. These chopped fiber-rovings fell onto a continuous resin mat in a production line and were then covered with another resin mat. Both surfaces were then covered with a protective foil. The material was rolled up afterwards [10].

**Figure 1:**
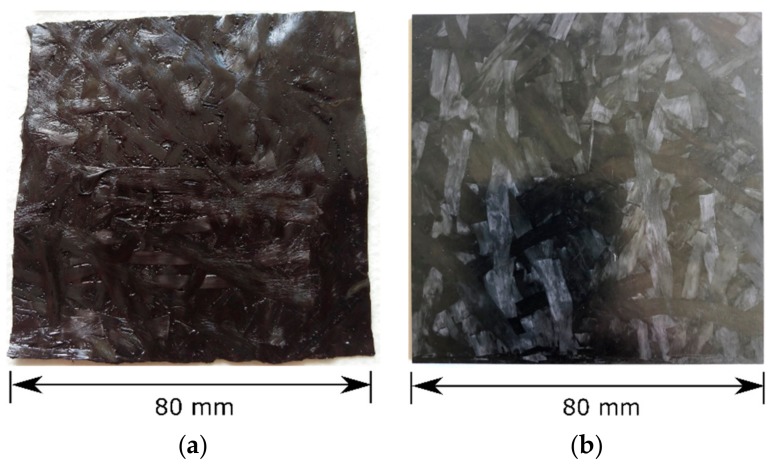
As-delivered (**a**) and pressed (**b**). C-SMC (carbon-fiber sheet molding compound) sheet: carbon-fiber sheet mold compound “Polynt SMCarbon 80 CF60-3K/2” [16].

To ease readability in the following, the acronym “C-SMC” is used for the material “Polynt SMCarbon 80 CF60-3K/2”. The material is shown in Figure 1 in delivery and pressed condition.

According to the datasheet 3000 filaments per roving were used and 50% of them were chopped into 25 mm length and 50% into 50 mm length. The C-SMC uses a fast curing epoxy resin. The cure cycle was 5 min at 145 °C and 100 bar internal mold pressure, according to the data sheet. Test plates with a thickness of 3.6 mm were produced with a covered mold area of 80% by the raw material, so material flow effects were kept on a low level. Every test plate was post cured at 160 °C for 2 h in an oven. The tensile modulus and strength were tested according to ISO 527-4, averaging 20 test specimens [17]. Table 1 lists the material properties of the mentioned C-SMC by means of mean value and one SD (standard deviation), if available. The glass transition temperature stated in the datasheet, was measured via DSC (differential scanning calorimetry). [18]

## 3. Experimental Procedure

### 3.1. Transverse Modulus in Thickness Direction

The elastic modulus in the thickness direction is necessary for modelling creep in the thickness direction. 

**Figure 2:**
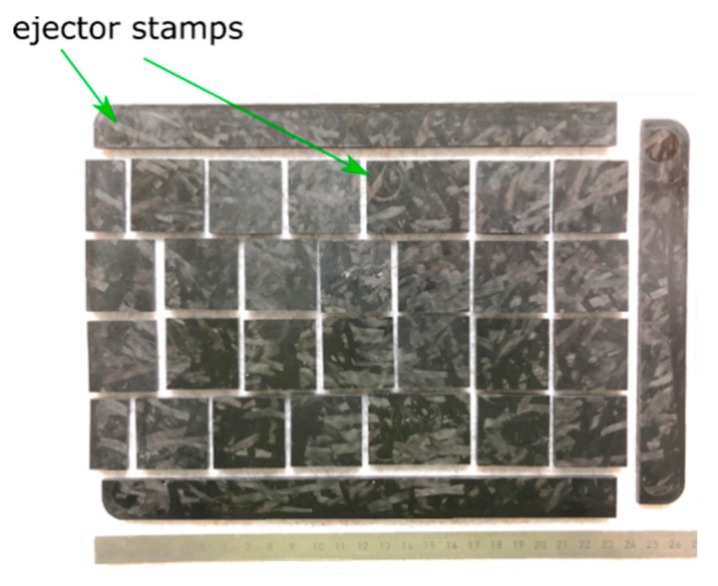
Preparation of SMC specimen out of the molded plate.

For this, C-SMC specimens with the dimensions 31.7 mm × 31.7 mm were prepared from a 3.6 mm thick coupon plate (Figure 2). A table buzz saw, with a CFRP-sawing blade, was used for cutting. Attention was paid to ensure that the test coupons were prepared with an even surface. Therefore, the areas with ejector stamp impressions were not used. Also, 15 mm of the sides were cut off due to turbulences of the fiber orientations.

**Figure 3:**
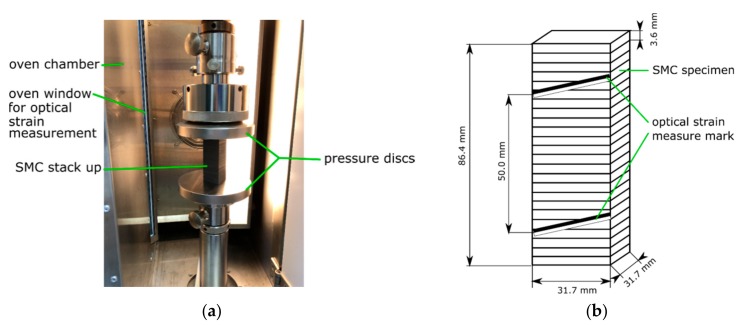
(**a**) C-SMC stackup between pressure discs in the universal testing machine; (**b**) C-SMC stackup in close-up view.

Twenty-four specimens were stacked, resulting in a 86.4 mm high stack. The stack was placed between pressure discs of a Zwick Roell 50 kN universal testing machine, equipped with an oven chamber (Figure 3). Before starting the test procedure, the specimens were stored without any preload in the oven at 120 °C for 12 h to ensure a constant temperature distribution.

For determination of the transverse modulus in thickness direction (z direction) the universal testing machine applied alternating forces between 5 kN and 15 kN ($F_{1}$ and $F_{2}$). The transverse modulus was calculated via formula 1. With A as the cross-sectional area of the test specimen, which is calculated to $31.7\;\mathrm{mm}\times 31.7\;\mathrm{mm}=1004{\;\mathrm{mm}}^{2}$. Strain variation of the stackup was tracked with the optical videoXtens system. $\varepsilon_{1}$ and $\varepsilon_{2}$ are the recorded strains at forces $F_{1}=5\;\mathrm{kN}$ and $F_{2}=15\;\mathrm{kN}$.

(1) $$E_{z}=\frac{\frac{F_{2}}{A}-\frac{F_{1}}{A}}{\varepsilon_{2}-\varepsilon_{1}}$$

**Figure 4:**
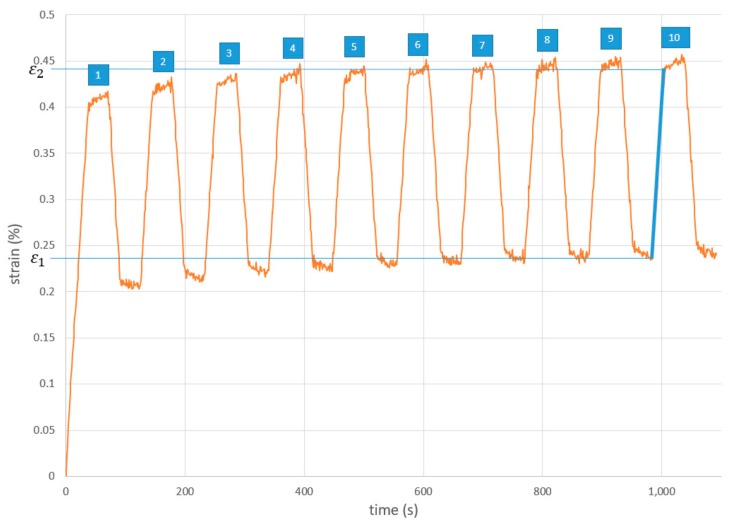
Alternating strains for determination of transverse modulus in z direction.

The alternating strains are shown in Figure 4. When a certain force level is reached, the strain does not remain constant, but shows a slight increase. This increase is due to the creep of the material, since the forces applied on the stackup are exclusively in the pressure range. The creep combines with the settling of the surfaces by the alternating forces to a steady increase of the absolute strain. After about 5 alternating force iterations, the steady increase in absolute strain is negligibly small (Figure 4). In order not to risk an erroneous increase of the measured modulus of elasticity, the modulus calculation was carried out in the 10th iteration. Values from the rise of 5 kN up until 15 kN are used for calculation of modulus. A transverse modulus of 5341 MPa in z direction at 120 °C is calculated.

The same procedure was conducted at room temperature, leading to a modulus of 5883 MPa. 

### 3.2. Compressive Creep Test

The same test setup as described in Section 3.1 was used for the compressive creep test. The universal testing machine was programmed to apply one specified force for 300,000 s (83.3 h). A force of 20,097 N was chosen for the first run and 2,009.7 N for the second run. Creep tests were always conducted on a before untested specimen stackup. The forces resulted in a constant stress in z direction of 20 MPa and 2 MPa. The level of stress was chosen, because the stress under the washers in the later described screw assembly was expected to be between 20 MPa and 2 MPa during most time of testing. There is no specification, which stresses should be used for curve fitting of the later described Norton–Bailey law. It is expected to have the highest accuracy, if the stresses at testing are on the same scale as the later simulated stresses. Measurement of creep strain was done via optical strain measurement (Figure 3). The machine was programmed to apply both stress levels within 60 s, because the manual performed tightening of a screw would take typically this time. 

**Figure 5:**
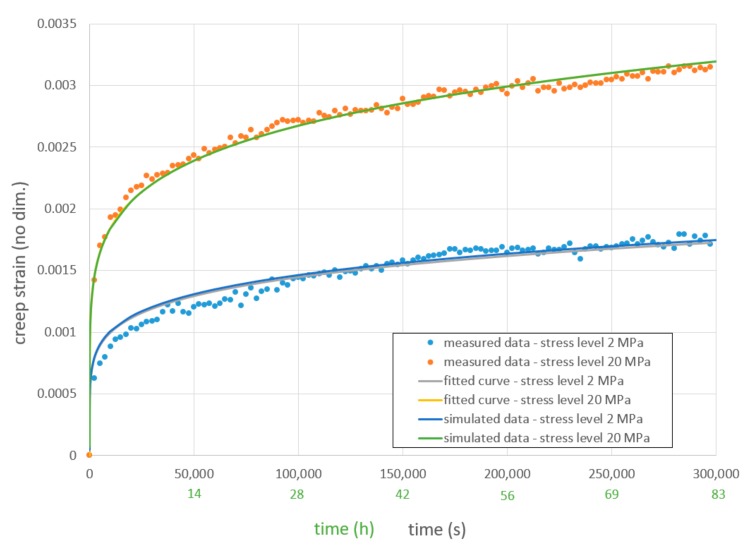
Measured, simulated (Section 4.1) and curve fitted creep strain–time graphs at different stress levels in z direction for “Polynt SMCarbon 80 CF60-3K/2” at 120 °C.

The strain measurement started immediately after the set force level was reached. Creep strain curves are shown in Figure 5 with a time resolution of 2500 s (0.69 h). The original recorded strains were negative, because pressure was applied. The sign of creep strain has been changed, since it is forbidden to use negative creep strain curves in the used FEA software SIEMENS NX Simcenter 12 [14]. 

The measurement revealed that the creep strain first increases sharply and then decreases significantly. This phenomenon is related to the general creep behavior, which is separated into primary, secondary, and tertiary creep. In primary creep stage the strain rate is much higher, compared to secondary creep. The gradient of secondary creep strain is almost constant and reaches a relatively low level, so-called minimum creep rate. The second creep stage lasts considerably much longer than the other two creep stages. Tertiary creep, which was not observed here, also has a much higher strain rate than the secondary creep stage. Creep mechanics at the first two stages finds its mechanical reasoning in thermal activated diffusion processes at atomic or molecular level. The atomic connections can rearrange in climbing or sliding movements, wherefore material strain increases over time. Not only this process, known as solid state diffusion, takes place, but also the polymer chains unfold through thermally activated diffusion. The first creep stage is accelerated in comparison to the second one, since there are still many atomic connections present, which need only a small amount of energy to rearrange. [19,20,21,22,23]

At tertiary creep, ongoing creep mechanisms from the secondary stage combine with local necking or cracks in the specimen. Thus, increased local stresses raise the creep strain rate in the tertiary stage [21,22,24].

The transition from primary creep to secondary creep takes place at around 50,000 s (13.9 h) at 2 MPa stress level and around 100,000 s (27.8 h) at 20 MPa stress level (Figure 5).

Development of a Norton–Bailey creep law approximation was the next step to fit the obtained data for the simulation. Three temperature dependent regression constants are needed for application of the power law. They can be determined by curve fitting of measured data via the method of least error squares. This method was used here, within a spreadsheet program. [8,25]

The Norton–Bailey creep law is shown in Equation (2). A, n, and m are regression constants, dedicated to one material at one temperature. The variable σ relates to the material stress in MPa, the variable t relates to the time in seconds. The variable $\varepsilon_{creep}$ is used for the creep strain, it should therefore be emphasized that no elastic strain portion of the initial compression is included here. It is necessary to use at least two stress levels (σ) for determination of n, which shifts the creep strain–time curve for different stress levels.

(2) $$\varepsilon_{creep}=A\times \sigma^{n}\times t^{m}$$

For the measured creep strain data (see Figure 5), the regression constants A=0.000187, n=0.267347, and m=0.161615 were determined via least error square method. These regression constants, used in Equation (2), resulted in a good approximation to original measured data (see ‘fitted curve’ in Figure 5). 

It should be noted that the obtained Norton–Bailey creep constants for the material are only valid, if the creep takes place in the first two creep stages. If the pressure is much higher than the 20 MPa mentioned above, a tertiary creep stage should be questioned, which would significantly accelerate the creep process. To rank at which pressure damage is to be expected in the laminate: A previous publication stated, that CFRP has a permissible surface pressure of 140 MPa or higher in static testing [2].

### 3.3. Preload Loss of Pretensioned Bolts

A screw assembly with a force measuring washer was set up to measure the loss of preload force over time at a real setup. The force measuring washer contains strain gauges, which are used to measure the applied compression force. Large-series steel washers had direct contact with the composite specimens with an inner and outer diameter of 9 and 24 mm (according to ISO 7093 [26]). A table buzz saw, with a CFRP-sawing blade was used to cut 55 mm wide square samples out of the 3.6 mm-thick coupon plates.

**Figure 6:**
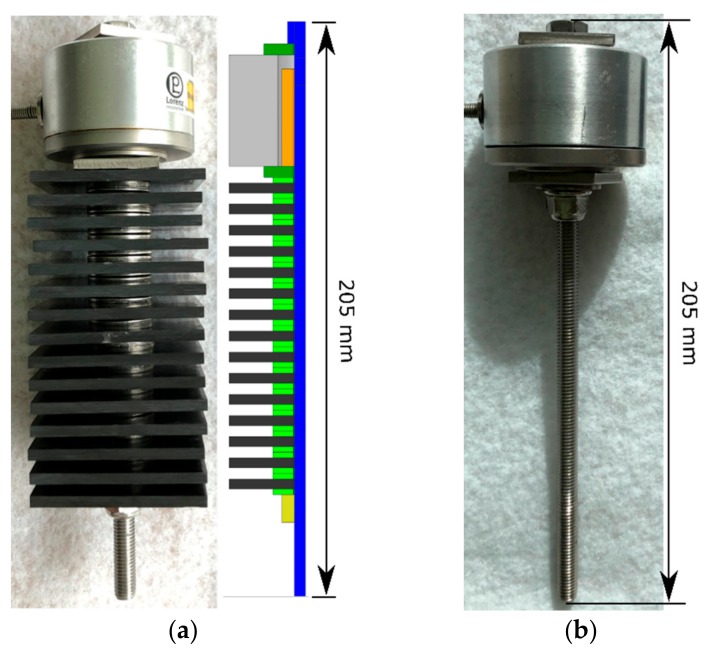
Screw assemblies for measurement of pretension loss; screw assembly (**a**) with 15 C-SMC specimens; screw assembly (**b**) with only steel clamped.

A 9-mm wide borehole was milled into the C-SMC specimens to have a loose fit with the M8 screw. The screw assembly consisted of 15 stacked specimens (screw assembly A). Every C-SMC plate was constrained with a washer at the top and on the bottom. The washers have one levelled side, which is placed in contact with the C-SMC coupons. (Figure 6)

All steel parts of the screw assembly are made from the stainless steel A2-70 with an expected $Rp_{0.2}$ of 450 MPa, which should be enough to keep the creepage of the metal on a negligible level [27,28].

In previous publications a correlation between the surface roughness of clamped parts and the loss of preload was stated [2]. A high surface roughness could lead to areas that are exposed to much higher stresses than the average stress under the washer. This could lead to increased creepage. Compressed molded parts usually have a high-quality surface, which often is untreated or just finished with some clear coating in the consumer market. No study was conducted in this paper to clarify the correlation between surface roughness and creep. For categorization and further studies, the surface roughness of the washer was measured to R_a_ = 1 µm and the C-SMC coupons to R_a_ ≤ 1 µm. 

The screw assemblies were stored without any preload force in an oven at 120 °C for 12 h to ensure a constant temperature distribution. Afterwards, the screws were tightened to 10 kN preload relatively fast (around 1 min) manually from outside of the oven. A wrench with extension was placed in a feedthrough, which is usually used to get wiring out of the oven. Inside the oven, a frame was placed, which is made from aluminum profiles, with a vice attached to it. The vice was holding the screw assembly. This test setup, where tightening of the screw can be done inside the closed oven, ensures a constant temperature through the assembly at all times of the experiment.

As mentioned, a previous publication stated that CFRP has a permissible surface pressure of 140 MPa or higher [2]. The M8 steel washer in the presented assembly has a contact area of 388 mm^2^ to the CFRP. With the preload of 10 kN a mean stress of 25 MPa was applied in z direction of the CFRP, when measurement started. Therefore, plastic deformation or damage in the C-SMC, due to too high preload on the screw, should not be an issue, following this rough estimation. 

**Figure 7:**
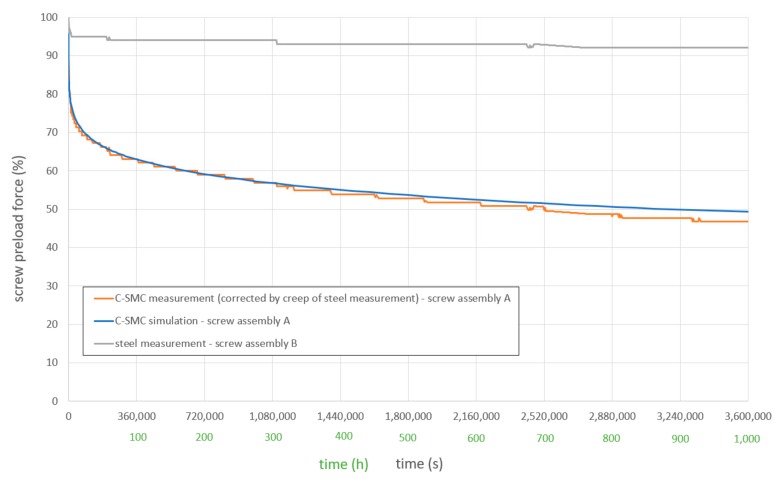
Loss of screw force over time.

The time resolved force changes of both screw assemblies A and B were tracked over 3.6 × 10^6^ s (1000 h). The change of the measured screw force over time is displayed in Figure 7. The loss of preload force of the steel specimens (screw assemblies B) was subtracted from the measured loss of preload of the C-SMC specimens. By this way, the data is cleared from creep of the force measuring washer itself. 

It can be seen, that screw force loss at screw assembly A is dominant in the primary creep stage within the first 360,000 s (100 h) and is then significantly reduced in the secondary creep stage. The graphs are staggered due to the discrete resolution of the measured screw preload force (Figure 7).

## 4. Simulation Procedure

### 4.1. Simulation of ‘Compressive Creep Test’

For testing of plausibility of the obtained data (Section 3.2) a simulation of the presented C-SMC stackup (Figure 3) was build up in SIEMENS NX Simcenter 12. For the most common commercial FEA software suits ABAQUS, Nastran, and ANSYS, no creep simulation is available for orthotropic materials, nor in SIEMENS NX Simcenter 12. This is problematic when it comes to prediction of creep behavior of composites. [29] 

The obtained regression constants from Section 3.2 and the transverse modulus in thickness direction from Section 3.1 were used to build up an isotropic material card for FE software. A Poisson’s ratio of 0.063 was used, which matches the zx Poisson’s ratio of a quasi-isotropic CFRP laminate. This value was calculated with the laminate calculation tool ICAN. The zx Poisson’s ratio was chosen, because the main load is applied in z direction by the screw. The zx Poisson’s ratio has the same value as the zy Poisson’s ratio in a quasi-isotropic laminate. The chosen fiber volume content was 50% [30].

The simulation was built up as a transient solution with solver 601. Fixed timesteps, which increase logarithmic over time, were defined. The logarithmic increase of the time step size seemed reasonable, since the strain changes at the beginning of the simulation are still greatest and will become smaller and smaller later. The first timestep sizes were only fractions of a second. 

The block was modeled solid and no contact formulations were used. Since there is a uniform stress state in all test coupons, contact modelling should not lead to a different result at all. The model contained a total of about 11,000 finite elements. Linear hexahedral elements were used.

The applied force was chosen as described in Section 3.2 to 20,097 N for the first simulation and 2009.7 N for the second simulation. The stress in the stackup was constant and the creep strain raised at every calculated timestep. The stress in z direction is analyzed here, because it is the biggest share of all occurring stresses and there is no significant difference to the effective stress distribution expected.

The effective stress, which is calculated with the deviatoric stresses, is used for the calculation of $\varepsilon_{creep}$ by the FEA solver (Equation (2)). The calculation is further explained in the “NX Nastran advanced nonlinear theory and modelling guide” [31].

The simulation revealed a good approximation to the fitted Norton–Bailey curve at both stress levels 2 MPa and 20 MPa (Figure 5). This can be seen self-evident on first sight, because the experiment from which the material data originates is simulated. But, the uncertain use of an isotropic material card, the determined transverse modulus and the calculated zx Poisson’s ratio were tested this way.

Another publication dealt also with the simulation of creep behavior of composites in the thickness direction [32]. An offset behavior between the simulated data and the measured data was observed there, using the FEA tool ABAQUS [12]. In response, a correction subroutine had to be developed. This procedure seems not to be necessary here, as the ‘simulated data’ curve in Figure 5 fits the ‘fitted curve’ precisely.

### 4.2. Simulation of ‘Preload Loss of Pretensioned Bolts’

**Figure 8:**
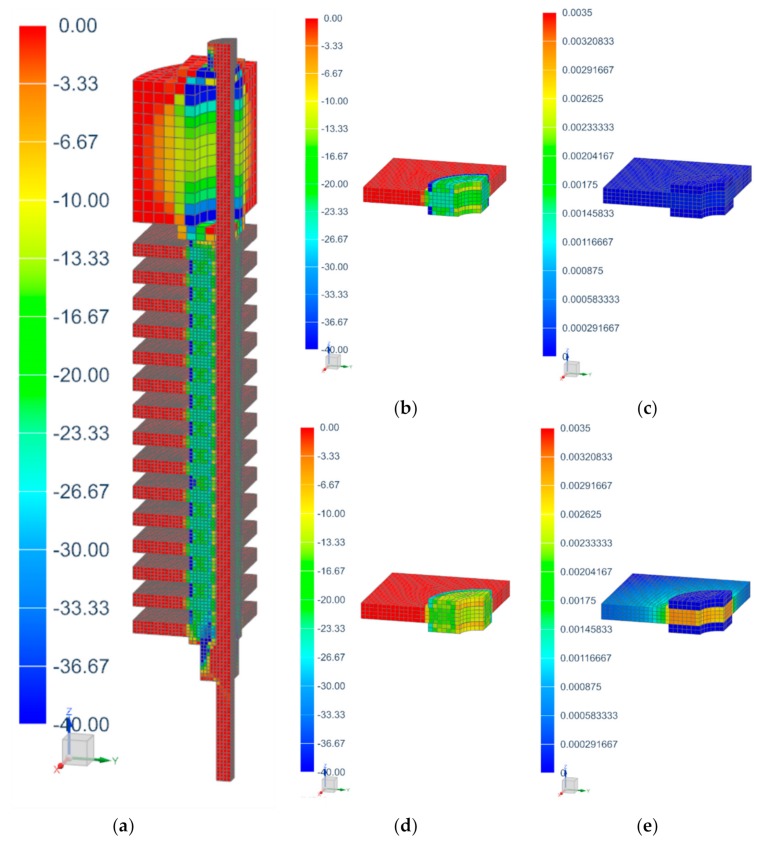
Simulation of screw assembly A in SIEMENS NX Simcenter 12 at 0.01 s (highest bolt pretension) and 300,000 s simulation time. (**a**) Stress in z direction at 0.01 s (MPa); (**b**) Stress in z direction at 0.01 s (MPa); (**c**) Creep strain at 0.01 s (no dim.); (**d**) Stress in z direction at 300,000 s (MPa); (**e**) Creep strain at 300,000 s (no dim).

A CAD (computer-aided design) (Figure 6) and FEA model (Figure 8) was created for the screw assembly to compare the measured preload loss with the developed creep model (Section 3.3). To achieve a faster calculation time, only a quarter of the assembly was modelled and then provided with symmetry conditions. Also, the stability of the nonlinear FEA is higher with a quarter model, according to experience. 

All contact areas of the screw assembly were provided with nonlinear contacts with a typical coefficient of friction of 0.15 between CFRP and steel. The coefficient of friction between metal and CFRP is discussed in various publications, e.g., [2,33], and varies from 0.05 up to 0.21. The selected value represents a compromise but should not have too much influence on the simulation result, since hardly any forces were transferred outside of the z direction in the contacts. The model contained a total of about 57,000 finite elements. Linear hexahedral elements were used. The simulated stress in z direction and creep strain of the C-SMC are shown in Figure 8. As expected, areas with stress concentrations revealed a higher creep strain increase over time. To be seen, for example, in the C-SMC under the washers. 

The simulation was built up as a nonlinear solution 601 in SIEMENS NX Simcenter 12. Fixed time steps, which increase logarithmic over time, were defined. The pretension of the screw was modelled by cooling of the screw neck and thus use of the material’s thermal contraction. In the first 0.01 s of the simulation, the screw neck was cooled down. A simple isotropic steel material card with 200 GPa Young’s modulus, Poisson’s ratio of 0.25, and a thermal expansion of 11 × 10^5^ 1/K was used for all steel parts. The C-SMC had the same material card, as described in Section 4.1. Heat transfers or temperature dependent material effects were not considered in this simulation. Thus, cooling of the screw neck was not affecting the CFRP specimen.

At 0.01 s a pretension of 2.5 kN, or respective 10 kN for a full model, was applied on the bolt. There was no larger time delay implemented, which could represent the actual tightening process, because measurement of creep strain also started just after the desired stress level was reached (Section 3.2). The bolt pretension in the simulation was evaluated via summarization of contact forces in z direction under the screw head. The progress of screw pretension loss over time, due to creep, is shown in Figure 7 by the graph ‘C-SMC simulation—screw assembly A’.

There is a high agreement between simulation and measurement data (Figure 7), even when creep data from previous measurement (Section 3.2) is extrapolated from 300,000 s (83.3 h) to a time forecast of more than 3,600,000 s (1000 h). 

### 4.3. Example of Application

There are different options for prevention of screw preload losses. When designing with polymers, the use of expansion bolts or conical spring washers is conceivable to compensate creep-induced preload losses. Both methods reduce the amount of preload loss, when the screw strain is lowering due to creep of the clamped material. Another possibility is to transfer the preload force of the screw not into the creeping material, but into an adapter sleeve made out of steel [34].

However, the last-named method is often entailed with higher implementation effort at part design and higher manufacturing costs. In the following, the first approach of compensating the bolt preload loss by conical spring washers is examined in more detail.

The obtained creep data from Section 3.2 was used for an exemplary screwed connection with a M8 screw and a large-series M8 washer, that clamps 4 mm of C-SMC on a steel block. Screw preload over time was compared to an assembly, which contains a conical spring washer according to DIN 6796 [35]. The screw simulation is methodically similar to the one in Section 4.2. 

**Figure 9:**
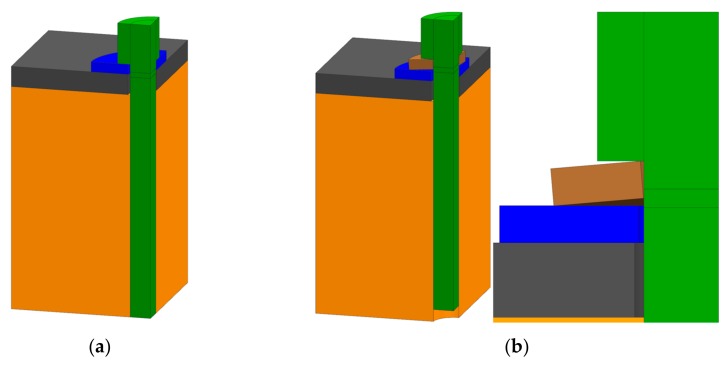
Application-oriented screw assemblies. (**a**) without conical spring washer. (assembly view); (**b**) with conical spring washer (assembly and detail view).

**Figure 10:**
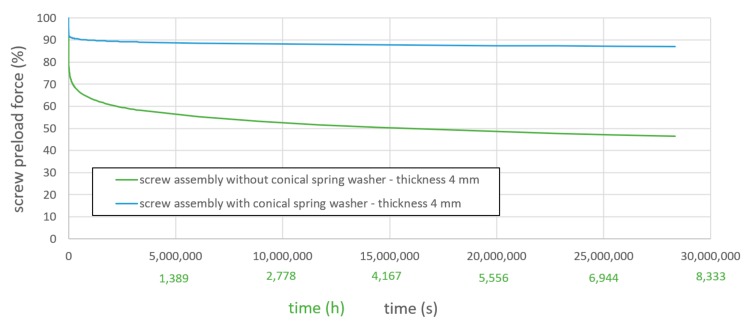
Simulated screw preload force over time of application-oriented screw assemblies.

The screws were tightened to 2.5 kN in the first 0.01 s of the simulation (Figure 9). The screw force of both assemblies was tracked over time and is shown in Figure 10. The graph ends at 2.8 × 10^7^ s (7777 h or 324 days).

It can be seen, that the screw assembly with the conical spring washer was able to maintain 87% of its preload force (8.7 kN) at the last time step, whereas the screw assembly without conical spring washer maintained only 46% (4.6 kN) of its original preload force. These values are not universal applicable for different screws, but have to be crosschecked at every use case. The percentage loss in preload force can be further reduced by using multiple spring washers in a bolted joint. The developed modelling approach proves its high effectiveness and enables long-term prediction of the reliability of the chosen screw design [36].

## 5. Conclusions and Outlook

This paper provides a simulative modelling approach of predicting the creep behavior of composite materials, that are clamped in screwed connections. 

A material card was created for the SMC material “Polynt SMCarbon 80 CF-60 3K/2” to simulate creep in thickness direction at 120 °C. A very good agreement was found between a bolt preload loss test and its simulation. The measured time of 300,000 s (83.3 h) as a basis for the Norton–Bailey creep law regression constants was not much, compared to a typical product lifetime. An extrapolation of the obtained data revealed a very good agreement even with a much longer run of the experiment of 3.6 × 10^6^ s (1000 h), which is a great advantage in terms of experimental effort. The test coupons for determining the Norton–Bailey regression constants as well as the screwed samples had a laminate thickness of 3.6 mm. It is expected, that the agreement between simulation and experiment is the highest, when both tests are done with the same laminate thickness. One carbon-fiber SMC with one material thickness was investigated in this paper. Testing more materials and different material thicknesses would be interesting aspects for future investigations. 

The lack of operability of the Norton–Bailey creep law in orthotropic material cards in current FEA software is a major drawback for composite material creep modeling. Nevertheless, as the main load at the screw assembly is applied in thickness direction, the FE simulation with an isotropic material card revealed still an accurate correlation with measured results (Figure 5 and Figure 7).

The high creep rate at the temperature of 120 °C was not expected for the investigated C-SMC. The phenomenon creep in thickness direction seems to be challenging for SMCs, even with a gap of 30 °C to the glass transition temperature. 

From design point of view, it was shown, that conical spring washers can be an effective way of limiting creep-induced loss of screw preload force.

## Figures and Tables

**Table 1:** Material properties of investigated C-SMC [18].

Material Property	Mean Value ± One SD
Modulus in-plane (measured)	46,765 MPa ± 2994 MPa
Modulus in-plane (datasheet)	46,000 MPa
Strength in-plane (measured)	312 MPa ± 16 MPa
Strength in-plane (datasheet)	240 MPa
Glass transition temperature (datasheet)	150 °C
Fiber weight fraction (datasheet)	60%
